# Testing the Simplified Molecular Dynamics Approach to Improve the Reproduction of ECD Spectra and Monitor Aggregation †

**DOI:** 10.3390/ijms25126453

**Published:** 2024-06-12

**Authors:** Attila Mándi, Aliz Rimóczi, Andrea Vasas, Judit Hohmann, Mahadeva M. M. Swamy, Kenji Monde, Roland A. Barta, Máté Kicsák, István Komáromi, Krisztina Fehér, Tibor Kurtán

**Affiliations:** 1 Department of Organic Chemistry, University of Debrecen, P.O. Box 400, 4002 Debrecen, Hungary; rimoczi.aliz@science.unideb.hu (A.R.); barta.roland@science.unideb.hu (R.A.B.); kicsak.mate@science.unideb.hu (M.K.); kurtan.tibor@science.unideb.hu (T.K.); 2 Doctoral School of Chemistry, University of Debrecen, Egyetem tér 1, 4032 Debrecen, Hungary; 3 Institute of Pharmacognosy, University of Szeged, 6720 Szeged, Hungary; vasas.andrea@szte.hu (A.V.); hohmann.judit@szte.hu (J.H.); 4 HUN-REN-USZ Biologically Active Natural Products Research Group, University of Szeged, Eötvös u. 6, 6720 Szeged, Hungary; 5 Faculty of Advanced Life Science, Hokkaido University, Kita 21, Nishi 11, Sapporo 001-0021, Japan; mswamy.madegowda@gmail.com (M.M.M.S.); kmonde@sci.hokudai.ac.jp (K.M.); 6 Vascular Biology, Thrombosis and Hemostasis Research Group, Hungarian Academy of Sciences, University of Debrecen, Nagyerdei krt. 98, 4032 Debrecen, Hungary; 7 HUN-REN–UD Molecular Recognition and Interaction Research Group, Egyetem tér 1, 4032 Debrecen, Hungary

**Keywords:** electronic circular dichroism, molecular mechanics, enhancement of agreement, aggregation

## Abstract

A simplified molecular-dynamics-based electronic circular dichroism (ECD) approach was tested on three condensed derivatives with limited conformational flexibility and an isochroman-2*H*-chromene hybrid, the ECD spectra of which could not be precisely reproduced by the conventional ECD calculation protocol. Application of explicit solvent molecules at the molecular mechanics (MD) level in the dynamics simulations and subsequent TDDFT-ECD calculation for the unoptimized MD structures was able to improve the agreements between experimental and computed spectra. Since enhancements were achieved even for molecules with limited conformational flexibility, deformations caused by the solvent molecules and multitudes of conformers produced with unoptimized geometries seem to be key factors for better agreement. The MD approach could confirm that aggregation of the phenanthrene natural product luzulin A had a significant contribution to a specific wavelength range of the experimental ECD. The MD approach has proved that dimer formation occurred in solution and this was responsible for the anomalous ECD spectrum. The scope and limitations of the method have also been discussed.

## 1. Introduction

In the calculation of chiroptical parameters, the conformational analysis step, consisting of the appropriate estimation of geometries and Boltzmann populations of low-energy conformers, is the key step to determine the absolute configuration and conformational ensemble with confidence [1,2,3,4]. There are ample examples reporting that relatively small changes in the geometry of the preferred conformer or in the populations of computed conformers can result in significantly different or near mirror-image electronic circular dichroism (ECD) spectra [5,6,7]. According to the generally applied protocol for the computation of ECD or other chiroptical data of small- or medium-molecular-weight derivatives, an initial conformational search is performed at a low level (usually molecular mechanics (MM) level implemented in different programs, e.g., Macromodel [8], Conflex [9] or Spartan [10]) as the first step to generate conformers systematically or by a specific algorithm [9,11,12]. Molecular dynamics (MD) offers an alternative way to generate the conformers at normal [13] or elevated temperature [14], which is however less utilized in chiroptical methods [13,15,16].

Molecular dynamics is able to simulate the natural motions of molecular systems by generating a trajectory of the molecule and thus it explores the molecular conformational hyperspace. During the calculation, successive geometries are generated by integrating Newton’s equations of motion, which enables the calculation of forces on atoms determined as derivatives of the potential energy. MD is a method of choice for the characterization of the structure and dynamics of biomolecules such as proteins [17,18], DNA [19] or carbohydrates [20,21] and it is also extensively used in material sciences [22]. MD is widely applied to refine experimental structures of biological macromolecules [23,24]. In addition, it is also possible to obtain macroscopic thermodynamic [25] and kinetic [26] properties from MD. This is enabled by the ergodic hypothesis stating that the time average of a property in a converged MD simulation equals the ensemble average of that property. Convergence of the simulation requires that the system reaches the equilibrium after exploring fully the conformational space, which means when the same conformations are visited repeatedly, the values of the properties no longer change.

MD can be used as a conformational search method by generating a large number of conformers [27]. However, large conformational barriers in the potential energy hypersurface can limit the full sampling of conformations available for the molecule as the simulation can only surpass energy barriers that are less or equal to the sum of the potential and kinetic energy of the molecules. Thermodynamic ensembles generated at higher temperatures or enhanced sampling methods can be used to overcome this limitation [28,29]. During NMR-based structure determination of biological macromolecules, MD implemented in a simulated annealing procedure is used to generate an ensemble of conformers, consistent with geometrical restraints derived from experiments [23,24].

When MD has been applied in chiroptical studies, it has been used for the verification or refinement of the conformational search results obtained by other methods [30,31,32]. Alternatively, it has been performed with the aim of exploring intermolecular interactions that were neglected by computing in vacuo or with a simple continuum model [16,33,34]. The accustomed utilization of MD conformers is similar to the ones obtained by the classical conformational search. They are optimized at a low (usually MM) level and then re-optimized at an advanced DFT level after clustering [13,14,15,33,35]. In order to address intermolecular interactions, which is not feasible with the classical ECD calculation approach, molecular complexes are sometimes extracted from the dynamics or generated manually [16,36,37,38]. While normal conformational searches are not able to address aggregates of the solute, MD simulations are widely used to study dimer or oligomer formation by aggregation of molecules of various sizes from small organic compounds up to nanoparticles or peptides [39,40,41,42,43,44,45]. In this respect, the MD-based ECD approach offers a great advantage compared to the classical ECD computation, which fails to treat efficiently weak intermolecular interactions resulting in the formation of dimers or higher aggregates. Whenever the ECD data cannot be attributed to a single molecule, since the aggregate formation markedly influences them, the MD approach prevails as a choice of method.

Recently, Grimme [46], Polavarapu [33] and their co-workers found that the omission of the MM and DFT re-optimization steps may lead to an improved agreement between the experimental and computed ECD data. According to this observation, the use of a dynamic abundance can have an advantage over a limited number of optimized local minima. Furthermore, geometrical consequences of the intermolecular interactions preserved in this way may also contribute to the improved reproduction of chiroptical spectra/properties, since Bannwarth et al. excluded the electronic effect of the solvent molecules in the ECD calculation step [46].

The application of explicit solvent molecules at high levels of theory during the DFT optimization and ECD calculation steps can be cumbersome since the more complex system requires more experience from the users and a significantly increased computational demand. Thus, we decided to test MD-based ECD methods in a simplified way, by applying explicit solvent models only in the MD step. In the following, we demonstrate the scope and limitations of this approach on the example of four previously studied derivatives (**1**–**4**) [30,47,48,49], for which only partial or borderline agreements could be obtained with the classical ECD computational analysis. Despite the low and moderate conformational flexibility of the studied molecules, the agreement could not be improved even by the application of several combinations of functionals and basis sets in both the DFT optimization and the ECD calculation steps. Herein, we present that the more diverse conformational ensembles generated by the MD-based conformational searches were able to improve the agreement of the computed ECD spectra with the experimental ones by using explicit solvation only in the MD step, and dimer formation can be responsible for anomalous ECD regions, which cannot be reproduced by considering a single molecule.

## 2. Results and Discussion

**Figure 1:**
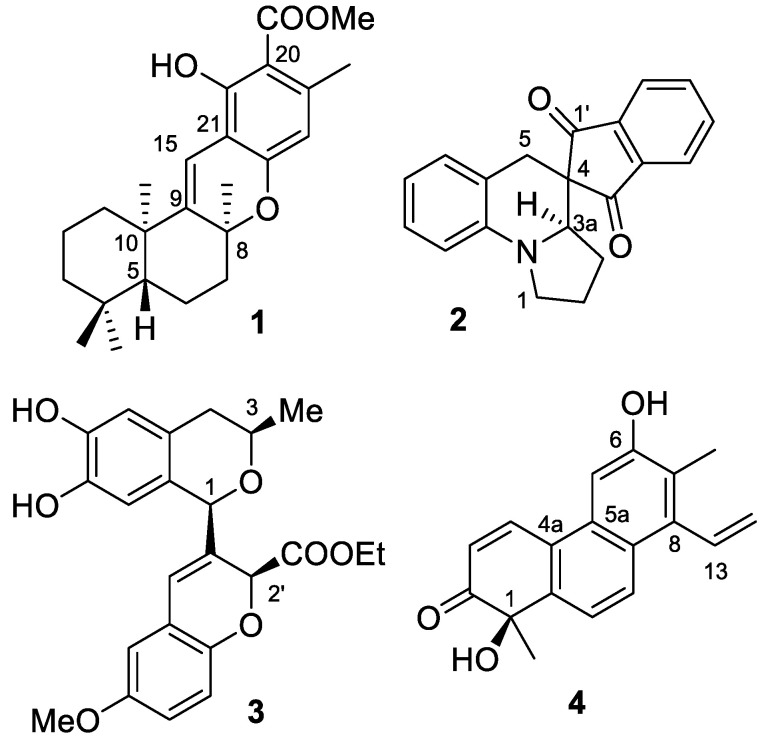
Structures of the studied molecules (−)-(5*R*,8*S*,10*R*)-9,15-didehydro-hongoquercin A methyl ester (**1**), (*R*)-**2**, (1*R*,3*R*,2′*S*)-**3** and (*R*)-luzulin A (**4**).

The conventional ECD computation and solid configurational assignment of the four selected molecules **1**–**4** (Figure 1) have already been reported by us in four separate papers [30,47,48,49]. Our current goal is to demonstrate how the MD approach can improve the imperfect agreement of the experimental ECD with the one computed by different combinations of the classical approach [2,3].

**Figure 2:**
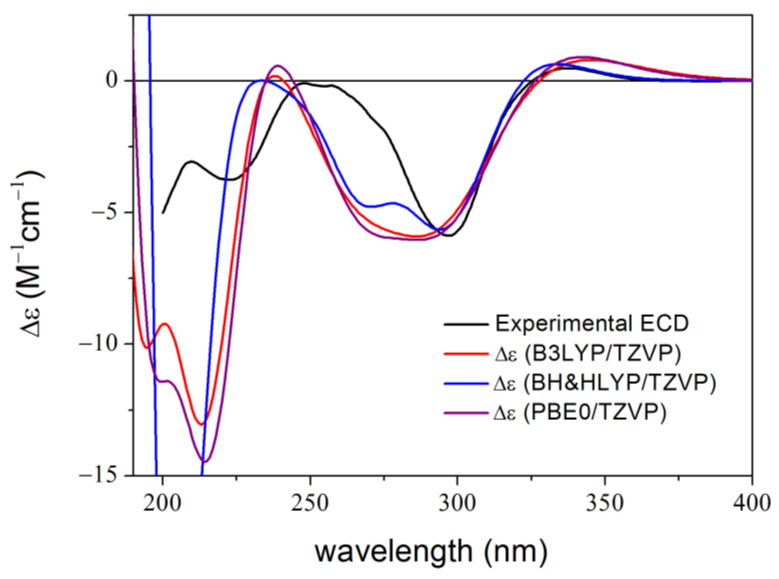
Experimental ECD spectrum of **1** in MeCN compared with the calculated spectra of (5*R*,8*S*,10*R*)-**1** at various levels with PCM for MeCN computed for the lowest-energy B3LYP/TZVP level PCM/MeCN optimized conformer (re-optimization of the Conflex MMFF94S conformers) [30].

The (−)-(5*R*,8*S*,10*R*)-9,15-didehydro hongoquercin A methyl ester (**1**) is a semi-synthetic derivative obtained from (+)-daurichromenic acid in an electrocyclization reaction [30]. It was an instructive example of how the derivatization of the conformationally flexible (+)-daurichromenic acid reduced the conformational freedom to less than 5% of the original with different combinations of conformational search packages and DFT optimizations [30,50]. A single major conformer with over 97% abundance was obtained at all the applied DFT levels, and reproduction of the major ECD transitions afforded the assignment of the absolute configuration (Figure 2). The shape, position and relative intensities of the low-wavelength ECD transitions (below 280 nm) obtained with the CS-based method, however, did not give a good agreement with the experimental data, especially considering that only a single major conformer was identified. In the experimental spectrum, the 299 nm negative ECD transition had a much smaller half-band width than those in the computed ones and the lower-wavelength computed transitions showed hypso- and hyperchromic shifts compared to the measured ones. DFT optimizations at different levels of theory followed by TDDFT-ECD calculations could not improve the agreement.

**Figure 3:**
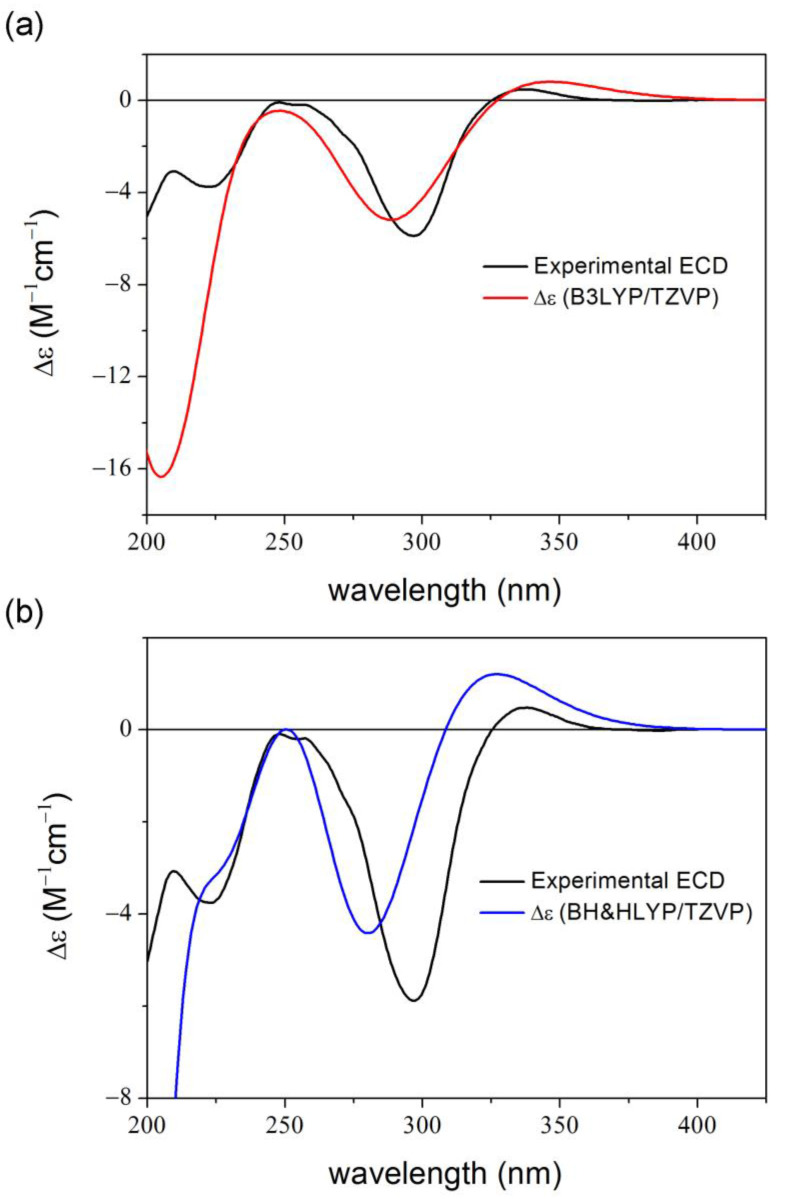
Experimental ECD spectrum of **1** compared with (**a**) the average B3LYP/TZVP PCM/MeCN spectrum of (5*R*,8*S*,10*R*)-**1** computed for 20 unoptimized snapshots taken from the 4 ns dynamics in MeCN; (**b**) the average BH&HLYP/TZVP PCM/MeCN spectrum of (5*R*,8*S*,10*R*)-**1** computed for 20 unoptimized snapshots taken from the 100 ns dynamics in MeCN.

When computing ECD spectra using the same TDDFT levels for 20 unoptimized MD conformers obtained equidistantly from a 4 ns simulation, the location and the relative intensity of the major transitions could be significantly enhanced, while it failed to reproduce the negative shoulder at 225 nm (Figure 3a). Since it is common to calculate ECD spectra at various levels of theories and present only the best match in case of similar results, we follow this approach for the MD-based results [1,2,51].

Besides the 4 ns dynamics, we also conducted a 100 ns dynamics run for (5*R*,8*S*,10*R*)-**1**, from which 20 structures were taken equidistantly similarly to the shorter dynamics. The average BH&HLYP/TZVP PCM/MeCN ECD spectrum of the 100 ns run showed a shoulder around 225 nm (Figure 3b), which was the only minor component missing from the ECD results of the shorter sampling. A larger number of trajectories (50 and 100) were also considered from the same dynamics but no further enhancement of the agreement could be achieved.

**Figure 4:**
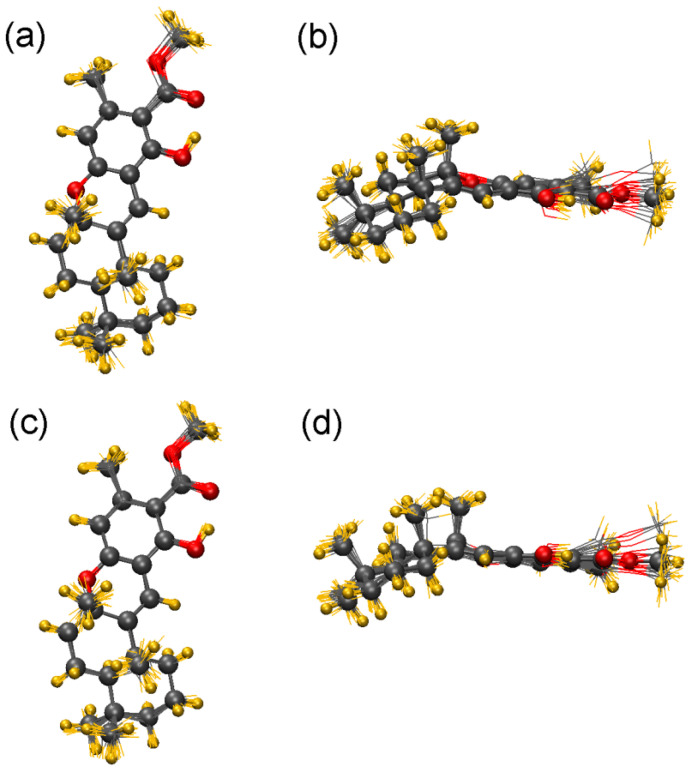
Comparison of the single low-energy B3LYP/TZVP PCM/MeCN conformer (CPK representation; grey: carbon, red: oxygen; yellow: hydrogen) with the 20 snapshots form the (**a**,**b**) 4 ns MD, (**c**,**d**) 100 ns MD (all MD structures with line representation) of (5*R*,8*S*,10*R*)-**1** utilized for the ECD calculations; (**a**,**c**) top view, (**b**,**d**) side view.

Compound **1** is a good example to demonstrate that insufficient reproduction of the experimental ECD spectrum, even in a solvent less characteristic to form molecular complexes with the solute, can be attributed to factors different from the wrong estimation of the Boltzmann-population of the local minimum conformers (every applied DFT level in the original study yielded a single major conformer) [30]. The MD-based ECD method performed without optimization can contain additional geometrical information, such as deformations induced by the solvent, which cannot be modeled properly with the classical TDDFT-ECD calculations performed in the gas phase or with continuum models (see Figure 4) [46].

**Figure 5:**
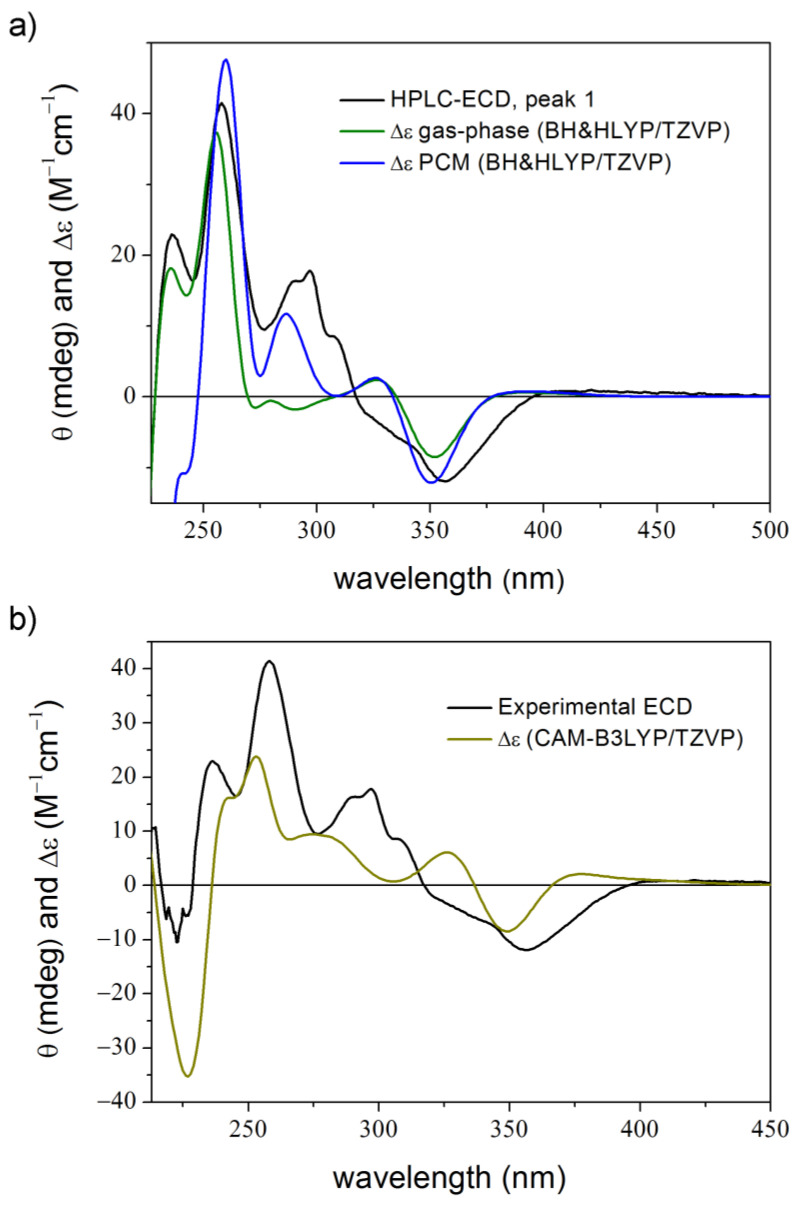
HPLC-ECD spectrum of the first eluting enantiomer of **2** (black line) compared with (**a**) the Boltzmann-weighted BH&HLYP/TZVP and BH&HLYP/TZVP PCM/CHCl_3_ TDDFT-ECD spectra of the low-energy (*R*)-**2** conformers in gas phase (green line) and PCM solvent model for CHCl_3_ (blue line). Levels of DFT optimization: B3LYP/6-31G(d) and B3LYP/TZVP PCM/CHCl_3_ of the MMFF conformers [47]; (**b**) the average CAM-B3LYP/TZVP PCM/CHCl_3_ TDDFT-ECD spectrum of the 100 unoptimized conformers taken from the 100 ns dynamics (olive line).

Compound **2** is a synthetic derivative obtained as a racemic mixture in a Knoevenagel-[1,5]-hydride shift-cyclization domino reaction and the ECD spectra of the enantiomers were measured with the online HPLC-ECD method [47]. According to the TDDFT-ECD studies, the first-eluting peak was found to be the (*R*) enantiomer. It was interesting that despite the low flexibility of the molecule with three initial MMFF conformers and two DFT conformers after re-optimization, no applied combination was able to reproduce all three major positive transitions at ca. 300, 260 and 240 nm. Only two of them were obtained in every case, although the main features of the experimental spectrum could be reproduced by the calculations (Figure 5a) [47]. Application of B97D or CAM-B3LYP functionals for the DFT optimization could not enhance the agreement achieved for the B3LYP conformers.

**Figure 6:**
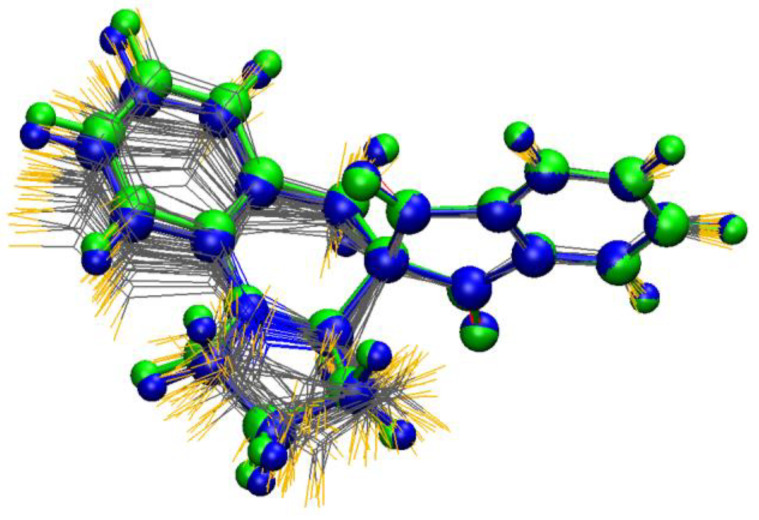
Lowest-energy B3LYP/6-31G(d) (green) and B3LYP/TZVP PCM/CHCl_3_ (blue) conformers of (*R*)-**2** from the classical approach [47] compared with the unoptimized conformers of the best matching MD region (41–80 ns) of the 100 ns dynamics run in CHCl_3_.

By applying the MD-based ECD technique, the 4 ns dynamics with 20 conformations gave similar results to the classical solvent-model calculations, while the 100 ns simulation with 100 conformations could reproduce also the 240 ns positive shoulder (Figure 5b). From the latter 100 MD structures, the 41–80 ns part reproduced best the experimental ECD spectrum (Figure S1) the geometries of which were compared to those of the lowest-energy ones resulting from the classical gas phase and solvent model approaches (Figure 6). The MD structures showed variations in the conformation of the five- and six-membered heterorings and thus the relative orientation of the benzene ring of the tetrahydroquinoline subunit also showed larger deviations, since it is condensed with the six-membered heteroring.

**Figure 7:**
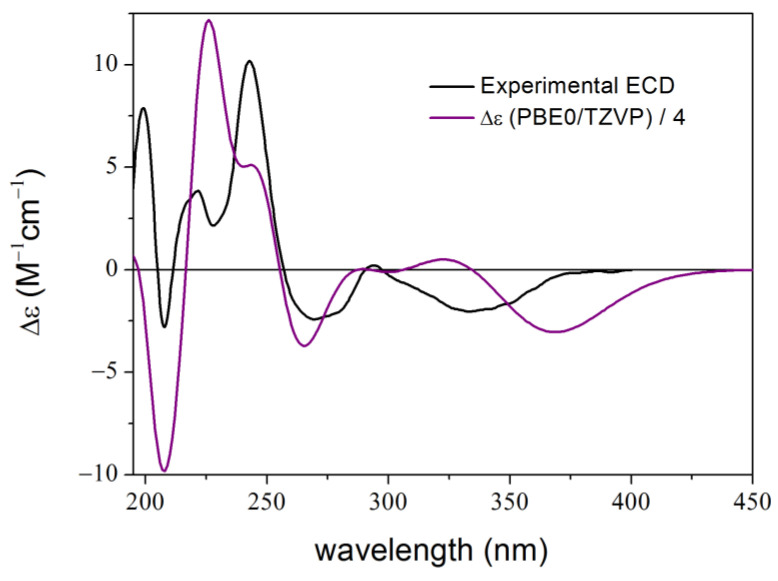
Experimental ECD spectrum of **3** (black curve) compared with the Boltzmann-weighted PBE0/TZVP (PCM/MeCN) ECD spectrum (purple curve) of (1*R*,3*R*,2′*S*)-**3** computed for the CAM-B3LYP/TZVP PCM/MeCN conformers (20 lowest-energy re-optimized MMFF conformers obtained with MacroModel) [48].

Compound **3** is a synthetic optically active isochroman-2*H*-chromene conjugate, which showed potent neuroprotective and anti-inflammatory activities [48,52]. Conventional solution TDDFT-ECD protocol performed at various combinations of levels could reproduce the sign of the major transitions but had serious difficulties in reproducing the relative intensities throughout the ECD spectrum and the location of the individual transitions in the high-wavelength region (Figure 7).

ECD spectra computed for the 4 ns MD could reproduce the shape and the location of the ECD transitions much better than the original approach (Figure S2). Interestingly, a long (100 ns) MD did not perform well in this case. By analyzing the long MD run, we found that the *M*-helicity of the heteroring of the isochroman unit was constant during the dynamics (only transiently changing into the other, *P*-helicity form) but the torsional angle of the C-1–C-3′ axis changed significantly around 23 ns (Figure S3), i.e., the orientation of the chromene subunit changed to opposite relative to the isochroman moiety. In contrast, all low-energy conformers in the original work had very similar torsion angles. Thus, the long dynamics ran into a high-energy region and the average conformation gained from this MD is far from the real one. Accordingly, the average RMSD of the 100 ns run was much higher than in the 4 ns run (Figure S4). The RMSD calculated for all carbon and oxygen atoms was ~3.0 Å after 23 ns in the long MD and ~0.7 Å before 23 ns and in the short MD. (The RMSD calculated for all atoms would be c.a. half of those computed for only the C and O atoms).

**Figure 8:**
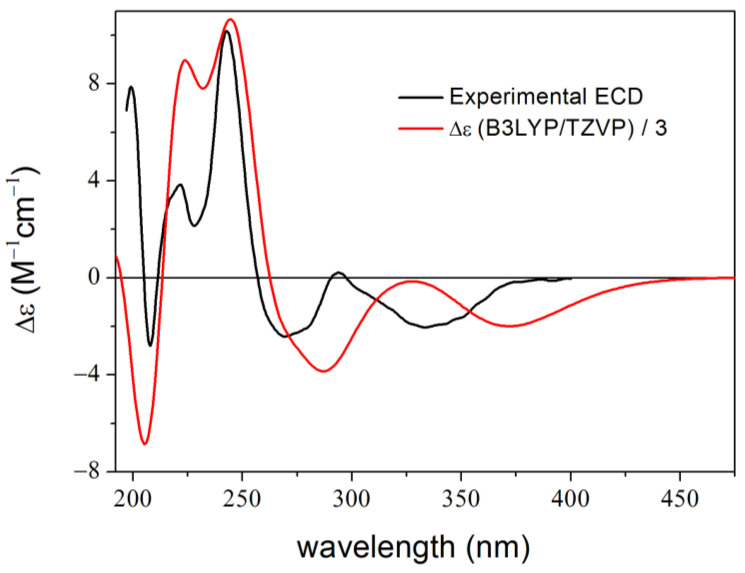
Experimental ECD spectrum of **3** and average B3LYP/TZVP PCM/MeCN spectrum of (1*R*,3*R*,2′*S*)-**3** computed for 100 unoptimized snapshots taken from the second (good) 100 ns dynamics in MeCN.

Then, we repeated the 100 ns dynamics, and this time the key torsional angles (Figure S5), the RMSD, and the ECD results (Figure 8) were similar to those of the short MD and the 1–23 ns part of the first 100 ns MD. (Unlike in geometry optimization, at the start of MD simulations the particles of the system are assigned random initial velocities, which influence the trajectory. This can also lead to the observation of rare events, however, due to the computational cost, MD simulations are limited to a certain timeframe, which may not always be sufficient to observe these rare events. In our case, we wanted to avoid such rare conformational changes, to get similar conformers to the classical approach). This is a good example that one should be careful by applying an MD-based method, and the conformational/ECD results should be validated with the classical approach.

**Figure 9:**
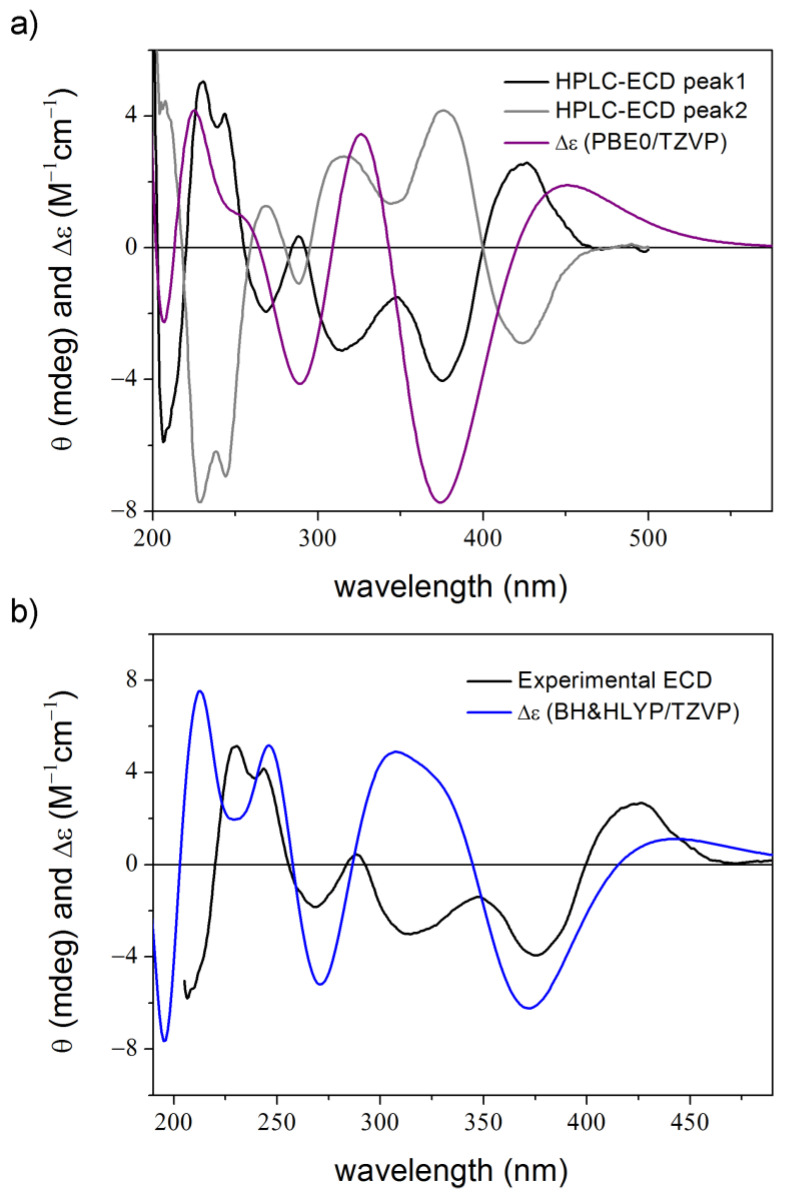
(**a**) Experimental HPLC-ECD spectra of **4** (black: first eluting enantiomer; grey: second eluting enantiomer) compared with the Boltzmann-weighted PBE0/TZVP (PCM/CHCl_3_) ECD spectrum of (*R*)-**4** computed for the B97D/TZVP PCM/CHCl_3_ conformers (five lowest-energy re-optimized MMFF conformers obtained with MacroModel) [49]. (**b**) Experimental HPLC-ECD spectrum of the first-eluting enantiomer of **4** and average BH&HLYP/TZVP PCM/CHCl_3_ spectrum of (*R*)-**4** computed for 100 unoptimized snapshots taken from the 100 ns dynamics in CHCl_3_.

Compound **4** is a natural product named luzulin A, which was isolated from *Luzula luzuloides* (Lam.) as a scalemic mixture [49]. After recording the HPLC-ECD spectra of the separated enantiomers, the absolute configuration was determined by TDDFT-ECD calculations (Figure 9a). The conventional conformational search-based method could reproduce the positive couplet above 350 nm and the low-wavelength region of the experimental ECD spectrum of the first-eluting peak but no applied combination was able to provide agreement for the 270–350 nm spectral range [49].

The ECD spectra computed for the 20 and 100 trajectories obtained from the 4 ns and 100 ns MD calculations, respectively, gave similar results to the original TDDFT-ECD study with only a minor improvement of the overall agreement and failed to reproduce the 312 nm negative CE in the disputed section (Figure 9b).

**Figure 10:**
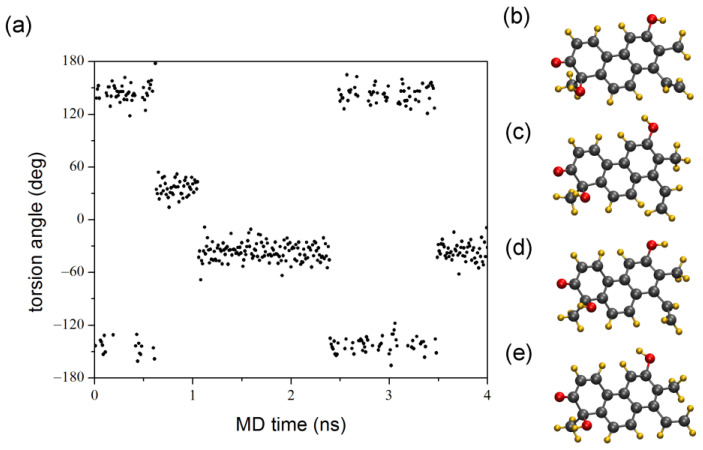
(**a**) Torsion angle profile of (*R*)-**4** along the C8-C13 bond (C8a-C8-C13-C14) during the 4 ns MD simulation with the representative structures of the individual states; (**b**) around +150°; (**c**) around +30°; (**d**) around −30° and (**e**) around −150°.

In order to reveal the origin of the discrepancy, the computed ECD spectra of the individual conformers in the original study and in the MD runs were reviewed but we could not find any conformers showing the correct ECD pattern in the 270–350 nm range. Compound **4** is quite a rigid molecule conformationally having flexibility only in the orientation of OH protons and the C-8 vinyl group. In the MD simulations, the average RMSD of the heavy atoms was around 0.35 Å in agreement with the rigidity of **4** (Figure S6). While rotation of the OH groups has a negligible effect on the ECD, the orientation of the vinyl group does have a considerable contribution (Figures S7–S9). However, this contribution appeared markedly below 300 nm, which alone could not explain the observed effect. The torsion angle profile for the rotation along the C-8–C-13 bond (Figure 10) indicated that even a short MD was sufficient to obtain all the possible conformer families of **4**, and thus a longer MD unfortunately could not considerably improve the overall ECD agreement in this case.

Then, we considered the possible complex forming effect of solvation with the polar component of the HPLC-ECD solvent mixture applied for the measurement [36,37]. To check this effect, a new conformational search was performed for (*R*)-**4** with an 84 kJ/mol energy window, and the OPLS_2005 [53] conformers were re-optimized at the ωB97X/TZVP PCM/CHCl_3_ level yielding the expected eight conformers differing in the orientation of the 6-OH and 8-vinyl groups (Figure S9). Then, isopropyl alcohol (*i*PrOH) was docked 20 times to each DFT conformer yielding nearly 3200 conformers of (*R*)-**4**–*i*PrOH complexes. The hits were merged and optimized at AM1 level first only for the hydrogen atoms and then for all atoms. Low-energy conformers in the first 13 kJ/mol range (326 conformers) were re-optimized at the ωB97X/TZVP PCM/CHCl_3_ level and ECD spectra were calculated for the conformers of the complex above 1% Boltzmann weight. The results were quite similar to those of the previous classical and MD-based approaches (Figure S10), indicating that the effect of the complex formation of **4** with the polar *i*PrOH component of the hexane/isopropyl alcohol 8:2 HPLC eluent is negligible for the ECD spectrum.

The aggregation of solute molecules is also known to have an impact on the ECD spectra since it is able to induce supramolecular chirality [54,55,56]. In 2018, Zając et al. investigated the aggregation of the red xanthophyll astaxanthin by conventional and MD-based TDDFT-ECD methods [57]. They could reproduce characteristic ECD transitions by calculations, which were missing from the computed spectrum of the monomer, while they were present in those of dimeric and decameric structures.

**Figure 11:**
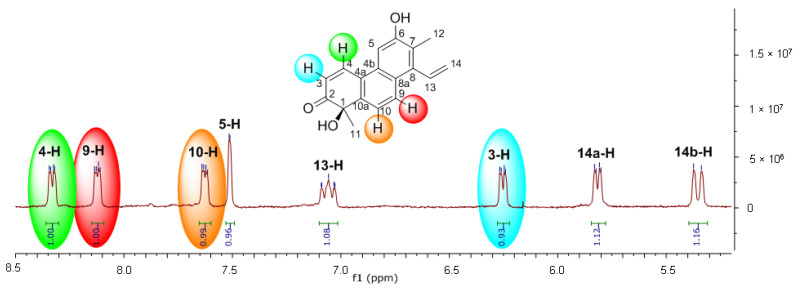
^1^H NMR spectrum of **4** showing broadening and duplications of signals.

By re-examining the experimental NMR data of **4**, we found experimental evidence for the aggregation. In the ^1^H NMR spectrum of **4**, characteristic proton signals were broadened and duplicated with a 1:1 ratio (Figure 11). The most intense broadenings could be observed for 3-H, 4-H, 9-H and 10-H indicating intermolecular hydrogen bonding of the C=O oxygen and π–π stacking interactions of the central aromatic ring.

**Figure 12:**
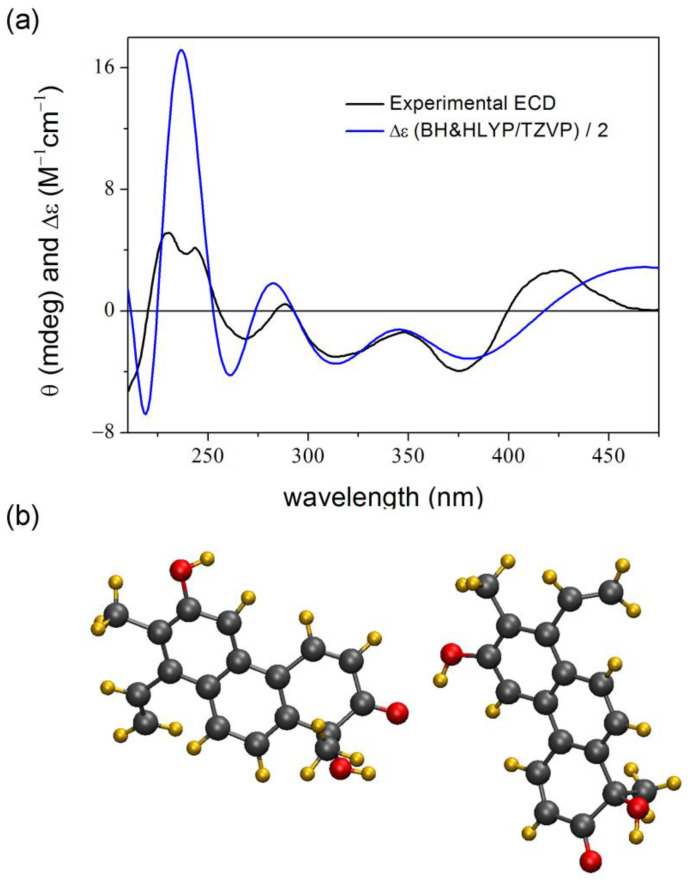
(**a**) Experimental HPLC-ECD spectrum of the first-eluting enantiomer of **4** compared with the BH&HLYP/TZVP PCM/CHCl_3_ spectrum of the best matching (*R*)-**4** dimer (7 ns) selected from the 100 ns dynamics run performed in CHCl_3_. (**b**) A dimer with one intermolecular hydrogen bond between the carbonyl and hydroxyl groups resulting the best match for the middle part of the ECD spectrum (7 ns).

In order to generate dimers, a chloroform box was built with two molecules of (*R*)-**4** placed at a distance of 20 Å from each other, and a 100 ns MD simulation was performed. Similarly to the monomer, 100 conformers were saved equidistantly and ECD spectra were computed for them. Visualization of the C-5a intermolecular distances during the 100 ns scan of the dimer (Figure S11) showed that dimers or other aggregates of **4** can form readily on a ns scale, which is in line with the experimental NMR proof for the aggregation. The average computed ECD spectra showed only a minor overall improvement compared to the MD results of the monomer, but we could identify a dimer in the 100 snapshots with excellent agreement, including also the problematic region (Figure 12). It is interesting that dimers of **4** with two or three intermolecular secondary interactions assuming stronger associations did not produce a better agreement of the ECD spectra. Thus, the best agreement was obtained for a dimer having only a single intermolecular hydrogen bond between the C-2 carbonyl oxygen and the C-6 hydroxyl group, while dimers with two or three intermolecular hydrogen bonds and π–π stacking had worse agreement (Figure S12).

**Figure 13:**
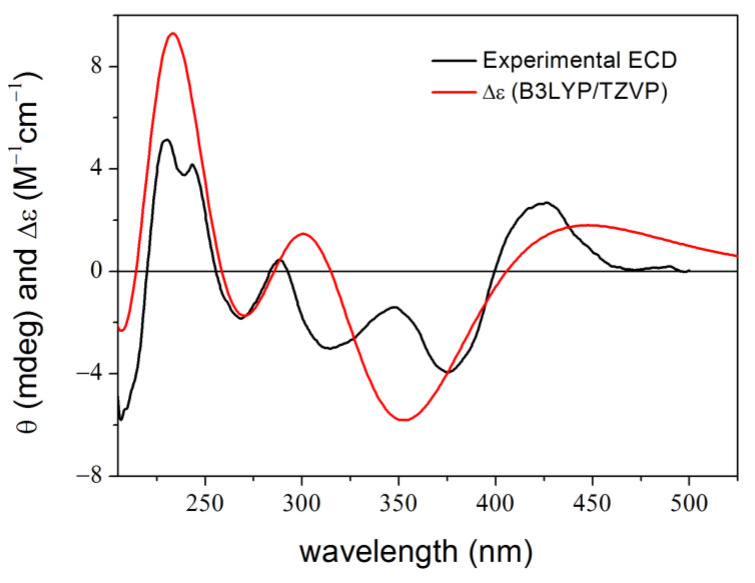
Experimental HPLC-ECD spectrum of the first-eluting enantiomer of **4** and average B3LYP/TZVP PCM/CHCl_3_ spectrum of two (*R*)-**4** molecules computed for 200 unoptimized snapshots taken from the 800 ns dynamics in CHCl_3_.

In order to improve the agreement, an 800 ns dynamics was performed for the same dimeric system, from which 200 snapshots were taken for the ECD calculations. The longer simulation time and the larger number of snapshots could improve the agreement for the disputed region (Figure 13). Now, the calculation reproduced all the major transitions and only separation of the 312 nm shoulder could not be observed from the 376 nm negative CE in the computed spectrum. An arbitrary re-weight of the conformers was also attempted by considering only the dimeric structures with less than 12 Å distance, but no further improvement could be achieved.

It is likely that instead of a prominent dimer, there is an ensemble of dimers, which is responsible for the observed ECD pattern in the 270–350 nm region, and the relative stability of the dimers has only a minor effect on the better agreement. The good estimation of the dimer–monomer dynamic equilibrium seems to be the key factor in enhancing the agreement in similar cases.

## 3. Materials and Methods

### 3.1. General Procedures

NMR spectra were recorded in CD_3_OD on a Bruker Avance DRX 500 spectrometer (Bruker, Billerica, MA, USA) at 500 MHz (^1^H). The signal of the deuterated solvent was taken as a reference.

### 3.2. Computational Section

#### 3.2.1. MD Simulations

Lowest-energy DFT conformers of the arbitrarily chosen enantiomers of **1**–**4** [30,47,48,49] were automatically parametrized by the antechamber module of AmberTools 1.5 in the GAFF force field [58], and solvated with a 16 Å layer of CHCl_3_ or MeCN using a cubic box. For CHCl_3_ the default CHCl_3_ box of the AmberTools was utilized, while for MeCN a box was created manually from parametrized MeCN molecules [59]. The cut-off used for non-bonded interactions was 9 Å. The particle-mesh Ewald procedure [60] was used to describe long-range electrostatic interactions. The SHAKE algorithm [61] was used to keep the bond lengths of hydrogen atoms rigid allowing a time step of 2 fs to be used. The MD simulations were carried out using AMBER simulation engine version 12 [14,62] using an NVT ensemble at 300 K. First, a minimization was performed for 10,000 steps starting with the steepest descent algorithm, which is switched to the conjugate gradient algorithm after 100 steps. After minimization a constant pressure MD was carried out for 0.4 ns with isotropic position coupling using a Berendsen barostat to equilibrate the system, during which the density stabilized, and the temperature settled at 300 K. Then, the pressure regulation was switched off and a classical constant total energy MD producing microcanonical NVE ensemble was carried out for 4 ns or 100 ns. The translational center of mass motions was removed every 1000 steps. AMBER 16 [63] and 22 [64] implemented on GPUs [65,66] were used to perform the 100 ns and the 800 ns simulations for the dimer of **4**.

#### 3.2.2. Analysis

Snapshots were saved into the trajectory every 1000 fs and 20, 100 or 200 out of these were chosen equidistantly for the ECD calculations. Torsion angles and RMSD were extracted from the trajectory using the cpptraj module [67]. For visualizing purposes, the VMD 1.9.2 [68], the PyMol 1.2r3pre [69] and the Molekel 5.4 [70] software packages were used.

#### 3.2.3. ECD Calculations

TDDFT-ECD calculations were run with various functionals (B3LYP, BH&HLYP, CAM-B3LYP, PBE0) and the TZVP basis set as implemented in the Gaussian 09 [71] and 16 [72] packages with PCM solvent model for CHCl_3_ or MeCN. ECD spectra were generated as sums of Gaussians with 1300–3600 cm^−1^ widths at half-height using dipole-velocity-computed rotational strength values [73]. All MD-based conformers were equally weighted.

#### 3.2.4. Docking and Solute–Solvent Complex Calculations

Mixed torsional/low-frequency mode conformational searches were carried out by means of the Macromodel 10.8.011 software by using the OPLS_2005 Force Field (MMFF) with an 84 kJ/mol energy window and an implicit solvent model for CHCl_3_ [8]. Geometry re-optimizations were carried out at the ωB97X/TZVP PCM/CHCl_3_ level. Isopropyl alcohol was docked 20 times to each ωB97X solute conformer by the AutoDock Vina 1.1.2 software [74]. For the docking, the energy window was set to 20 kcal/mol, exhaustiveness to 16, and a maximum of 1000 hits to save. In all docking runs, 19–20 hits were found. The hits were merged, and hydrogen was added with the OpenBabel 3.00 software [75], optimized at the AM1 level first only for the hydrogen atoms and then for all atoms. The lower-energy (first 13 kJ/mol) conformers were re-optimized at the ωB97X/TZVP PCM/CHCl_3_ level and ECD spectra were calculated for the conformers of the complex above 1% Boltzmann weight at the same four levels as for the MD-based structures.

## 4. Conclusions

In conclusion, four small organic molecules showing problematic calculated ECD spectra and low or moderate conformational flexibility were selected and tested by the novel simplified MD-based ECD method in two organic solvents. In all cases, at least a similar agreement could be achieved with all applied settings as in the original studies and in three cases considerable improvement of the ECD agreement could be achieved in some regions or in the whole spectrum with the optimized MD length and conformer number.

A relatively short 4 ns molecular dynamics simulation with a low number of conformers (20) performed on a single solute molecule was sufficient to improve the agreements in a rigid (**1**) or a medium flexibility case (**3**), while a longer MD with a larger number of conformers could improve the results in case of **2**. One has to be aware, however, of the unexpected direction of the MD. The MD method has to be validated with the classical conformational search-based approach and it is aimed primarily at enhancing the agreement by taking into consideration some deformational effects caused by the solvent, which cannot be modeled in the gas phase and continuum model simulations. The simplified MD-based approach may be less effective in increasing the agreement if stronger interactions with the solvent are present yielding complex formation.

For compound **4**, MD simulations confirmed that dimer formation occurred in solution and this was responsible for the anomalous ECD spectrum. The MD-based TDDFT-ECD spectra of the aggregate gave considerably better agreement than the classical approach, and a selected MD dimer could reproduce well the anomalous ECD region. For **4**, aggregation contributed significantly to the ECD spectrum and simplified treatment of the dimers was enough to considerably enhance the agreement, which was not possible by considering only a single molecule or solute–solvent complexes.

As is generally true in computational chemistry, it is worth considering the ratio of the invested work and computational resources vs. the expected result. The herein-tested simplified MD-based ECD method applies explicit solvent molecules only in the MD simulation, the next TDDFT step is already performed on the solute molecules, where the effect of the solvents is preserved via the unoptimized structures. In case one would like to further enhance the match of the experimental and calculated spectra, QM/MM or pure QM treatment of solute–solvent complexes gained from normal MD or methadynamics simulations could be considered for the TDDFT step, proper parametrization of the solute and solvent molecules could be applied instead of the automatic one, or other approaches for the generation of solute aggregates can be attempted as described in the literature, e.g., for the chiroptical studies of pantolactone or bilirubin and biliverdin analogs [76,77,78,79].

## Data Availability

Data is contained within the article and Supplementary Material.

## Supplementary Materials

The following supporting information can be downloaded at: https://www.mdpi.com/article/10.3390/ijms25126453/s1.
