# Supplementary material for: Testing the Simplified Molecular Dynamics Approach to Improve the Reproduction of ECD Spectra and Monitor Aggregation
Source: Int J Mol Sci. 2024 Jun 12;25(12):6453. doi: 10.3390/ijms25126453 (PMC11204327; doi:10.3390/ijms25126453)
Supplement: Supplementary file 1 [file ijms-25-06453-s001.zip › ijms-3009673-supplementary.pdf]

Supporting Information

Testing the Simplified Molecular Dynamics Approach to Improve the Reproduction of ECD Spectra and Monitor Aggregation

Attila Mándi [1,+,*], Aliz Rimóczi [1,2,+], Andrea Vasas [3,4], Judit Hohmann [3,4], Mahadeva M. M. Swamy [5], Kenji Monde [5], Roland A. Barta [1,2], Máté Kicsák [1], István Komáromi [6,†], Krisztina Fehér [7,*] and Tibor Kurtán [1]

[1] Department of Organic Chemistry, University of Debrecen, P.O. Box 400, 4002 Debrecen, Hungary
[2] Doctoral School of Chemistry, University of Debrecen, Egyetem tér 1, 4032 Debrecen, Hungary
[3] Institute of Pharmacognosy, University of Szeged, 6720 Szeged, Hungary
[4] HUN-REN-USZ Biologically Active Natural Products Research Group, University of Szeged, Eötvös u. 6, 6720 Szeged, Hungary
[5] Faculty of Advanced Life Science, Hokkaido University, Kita 21, Nishi 11, Sapporo 001-0021, Japan
[6] Vascular Biology, Thrombosis and Hemostasis Research Group, Hungarian Academy of Sciences, University of Debrecen, Nagyerdei krt. 98, 4032 Debrecen, Hungary
[7] HUN-REN–UD Molecular Recognition and Interaction Research Group, Egyetem tér 1, 4032 Debrecen, Hungary
[*] Correspondence: mandi.attila@science.unideb.hu (A.M.); feher.krisztina@science.unideb.hu (K.F.); kurtan.tibor@science.unideb.hu (T.K.)
[+] These authors contributed equally to this work.
[†] Deceased

Table of Contents

Figure S1. 3
Figure S2. 3
Figure S3. 4
Figure S4. 5
Figure S5. 6
Figure S6. 7
Figure S7. 7
Figure S8. 8
Figure S9. 9
Figure S10. 10
Figure S11. 10
Figure S12. 11

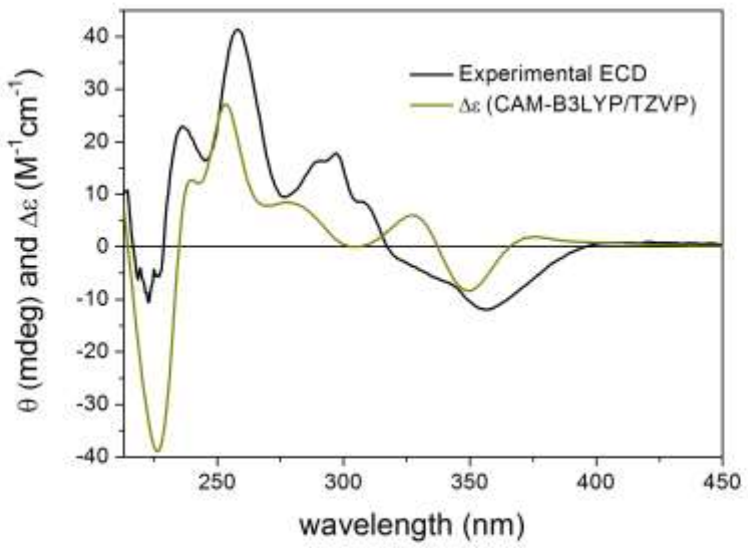

Figure S1. Experimental HPLC-ECD spectrum of the first-eluting enantiomer of **2** (black line) and average CAM-B3LYP/TZVP PCM/$CHCl_3$ spectrum of (*R*)-**2** (olive line) computed for 40 unoptimized snapshots taken from the 100 ns dynamics in $CHCl_3$ (best matching region of 41-80 ns).

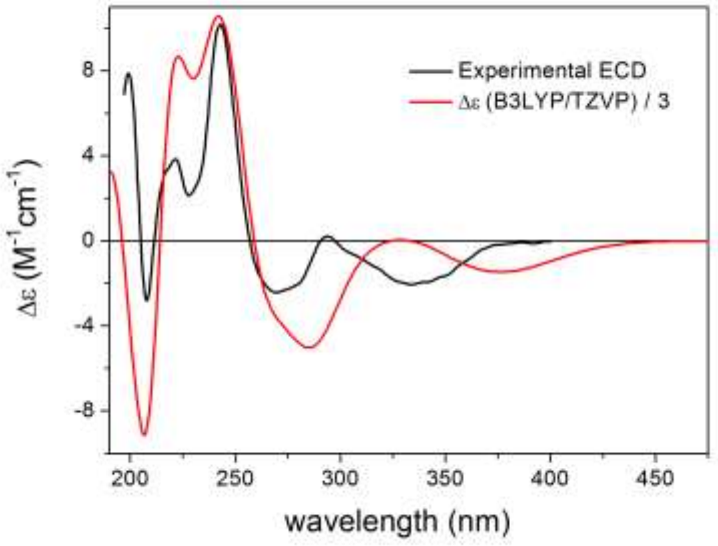

Figure S2. Experimental ECD spectrum of **3** (black curve) and average B3LYP/TZVP PCM/MeCN spectrum of (1*R*,3*R*,2'*S*)-**3** computed for 20 un-optimized snapshots taken from the 4 ns dynamics in MeCN.

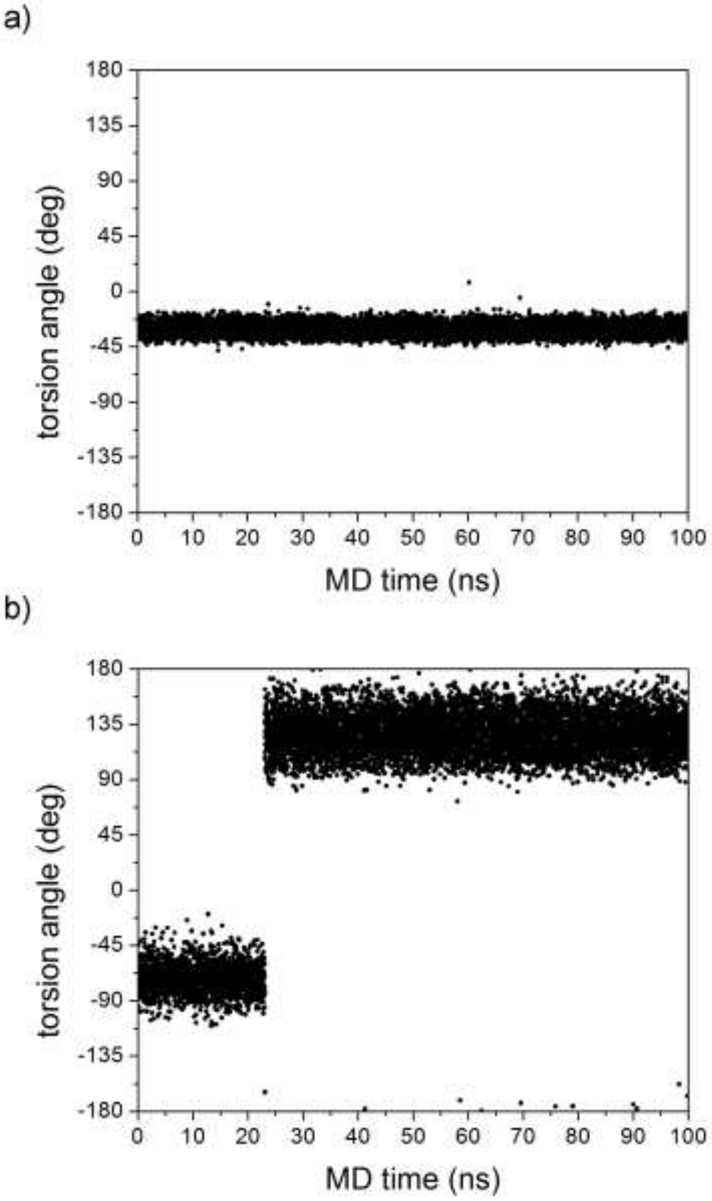

Figure S3. Time evolution of a) helicity of the heteroring of the isochroman unite of (1*R*,3*R*,2’*S*)-**3** during the first (wrong) 100 ns simulation (the torsion angle for helicity was defined by the atoms C4a–C8a–O2–C3); b) the torsion angle along the bond C1–C3’ (C8a–C1–C3’–C2’) of (1*R*,3*R*,2’*S*)-**3** during the 100 ns simulation.

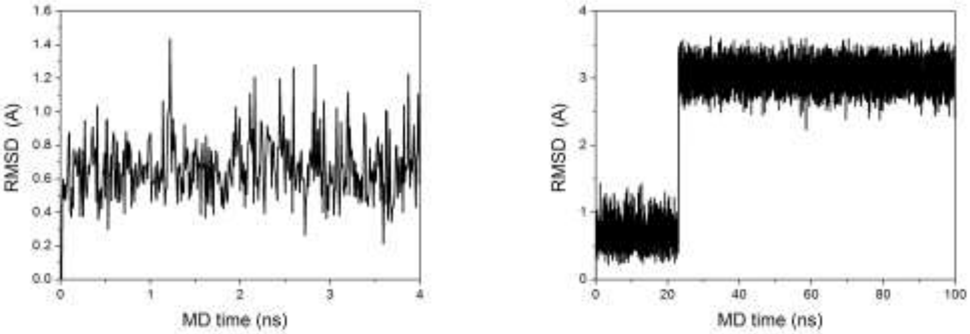

Figure S4. Time evolution of RMSD of the atomic coordinates for (1*R*,3*R*,2’*S*)-**3** in the short (4 ns, left) and the long (100 ns, right) MD runs computed for all C and O atoms.

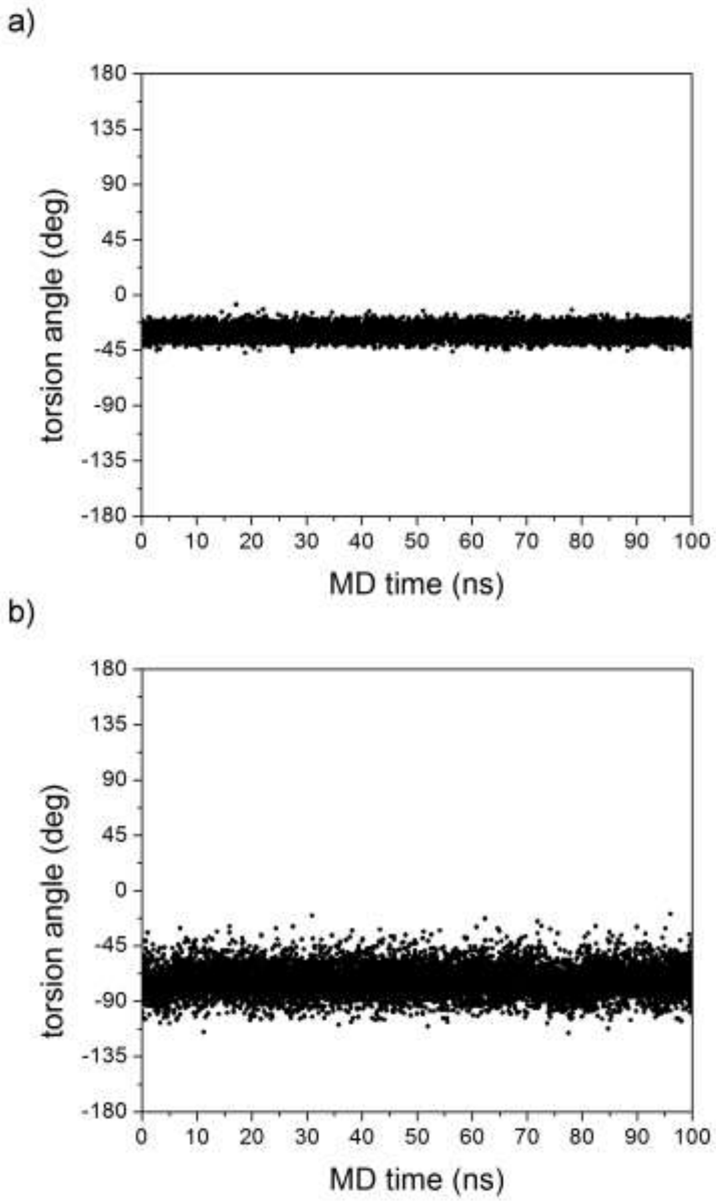

Figure S5. Time evolution of a) helicity of the heteroring of the isochroman unite of (1*R*,3*R*,2'*S*)-**3** during the second (good) 100 ns simulation (the torsion angle for helicity was defined by the atoms C4a–C8a–O2–C3); b) the torsion angle along the bond C1–C3' (C8a–C1–C3'–C2') of (1*R*,3*R*,2'*S*)-**3** during the 100 ns simulation.

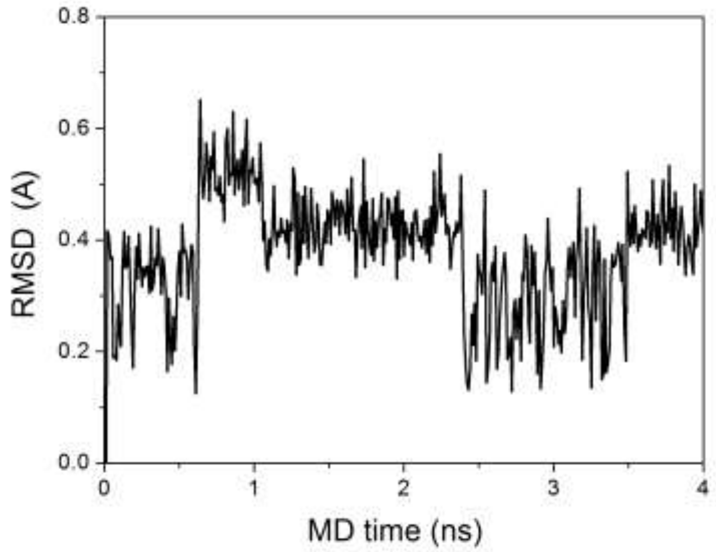

Figure S6. Time evolution of RMSD of atomic coordinates for (*R*)-**4** during the 4 ns molecular dynamics computed for all C and O atoms.

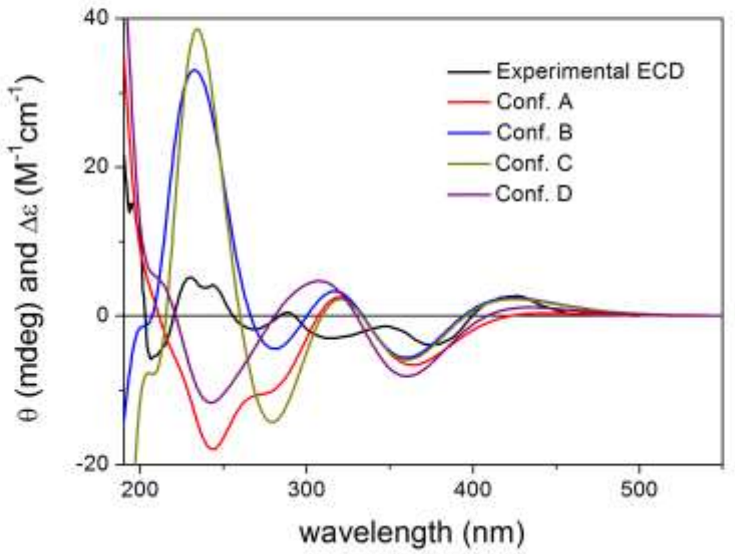

Figure S7. Experimental HPLC-ECD spectrum of the first-eluting enantiomer of **3** compared with the individual PBE0/TZVP PCM/$CHCl_3$ ECD spectra of the first four conformers of (*R*)-**3** computed for the ωB97X/TZVP PCM/$CHCl_3$ conformers indicating the effect of the orientation of the C-8 vinyl group.

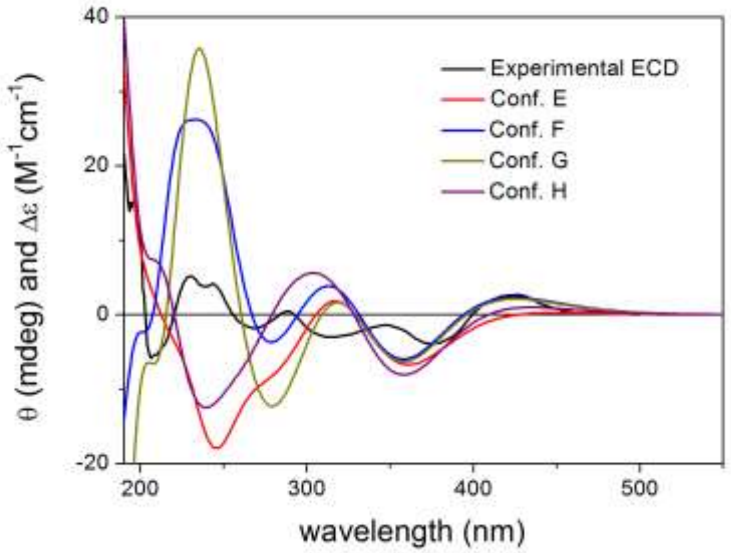

Figure S8. Experimental HPLC-ECD spectrum of the first-eluting enantiomer of **3** compared with the individual PBE0/TZVP PCM/$CHCl_3$ ECD spectra of the second four conformers of (*R*)-**3** computed for the ωB97X/TZVP PCM/$CHCl_3$ conformers indicating the effect of the orientation of the C-8 vinyl group and that of the 6-OH group in comparison with Figure S7.

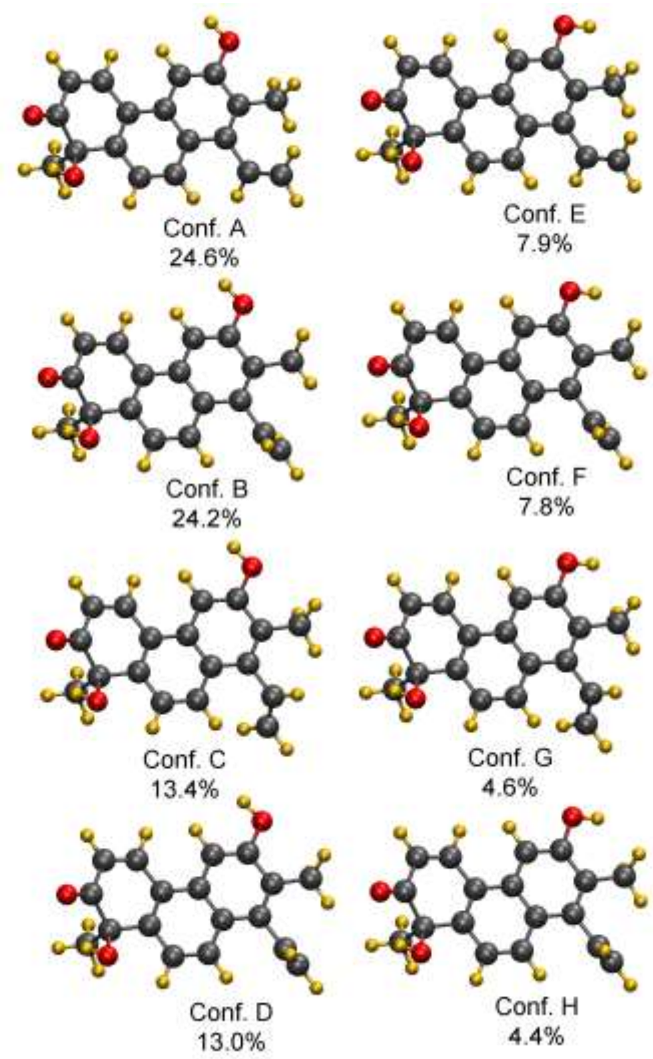

Figure S9. Low-energy conformers of (*R*)-**3** computed at the ωB97X/TZVP PCM/$CHCl_3$ level of theory. Conformers A and E would fit into group 4 represented by structure e on Figure 10; conformers B and F would fit into group 1 represented by structure b on Figure 10; conformers C and G would fit into group 2 represented by structure c on Figure 10; conformers D and H would fit into group 3 represented by structure d on Figure 10.

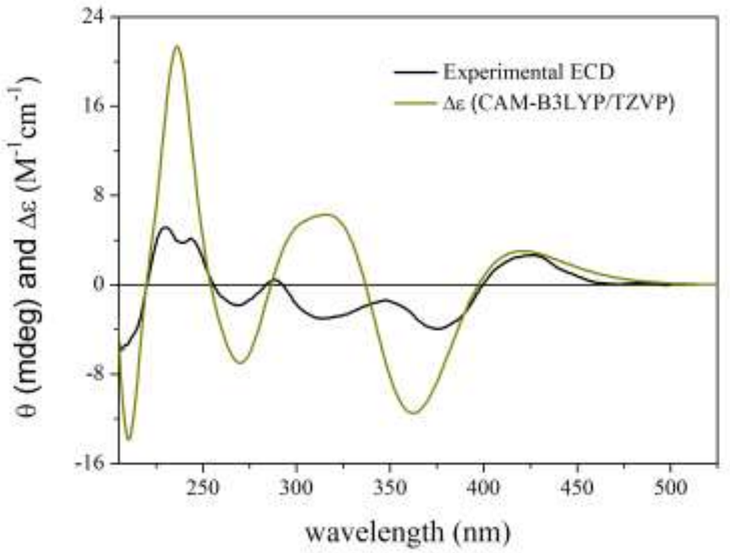

Figure S10. Experimental HPLC-ECD spectrum of the first-eluting enantiomer of **3** compared with the Boltzmann-weighted CAM-B3LYP/TZVP PCM/$CHCl_3$ ECD spectrum of the (*R*)-**3** – iPrOH complex computed for the ωB97X/TZVP PCM/$CHCl_3$ conformers (29 lowest-energy re-optimized AM1 conformers).

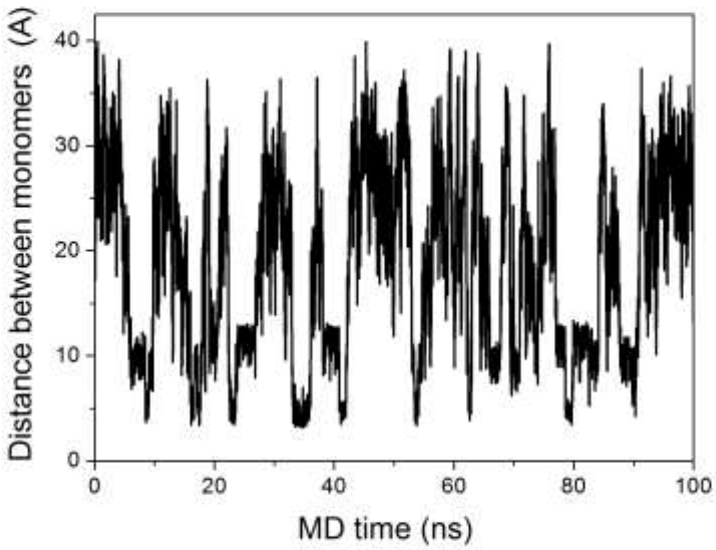

Figure S11. Distance between the monomers (considering the C-5a atoms) of (*R*)-**4** during the 100 ns MD simulation.

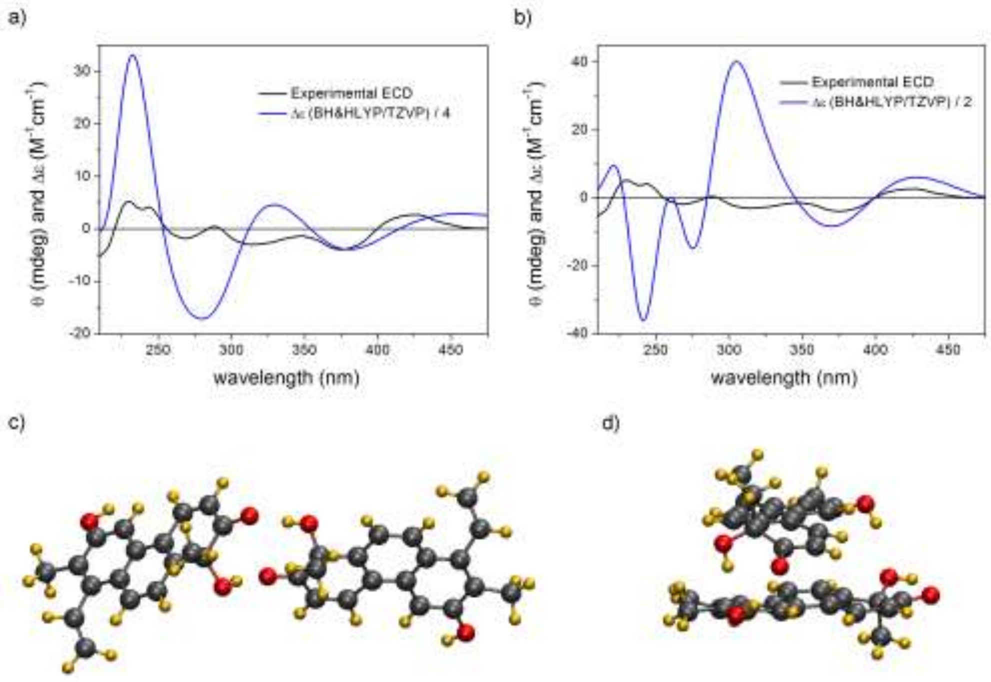

Figure S12. Experimental HPLC-ECD spectrum of the first-eluting enantiomer of **4** compared with the BH&HLYP/TZVP PCM/$CHCl_3$ spectrum of the (*R*)-**4** dimer taken at a) 24 ns and b) 34 ns from the 100 ns dynamics run in $CHCl_3$. Corresponding dimer structures of (*R*)-**4** from the 100 ns dynamics: c) a dimer with two intermolecular hydrogen bonds (24 ns) and d) a dimer with three hydrogen bonds and π-π stacking (34 ns).
